# "Enhanced" interrogation of detainees: do psychologists and psychiatrists participate?

**DOI:** 10.1186/1747-5341-3-21

**Published:** 2008-09-25

**Authors:** Abraham L Halpern, John H Halpern, Sean B Doherty

**Affiliations:** 1 New York Medical College, USA; 2 Laboratory for Integrative Psychiatry, McLean Hospital, Belmont, MA, USA; 3 Cardiff University, School of Medicine, Wales, UK; 4 McLean Hospital, 115 Mill Street, Belmont, MA 02478-9106, USA

## Abstract

After revelations of participation by psychiatrists and psychologists in interrogation of prisoners at Guantánamo Bay and Central Intelligence Agency secret detention centers, the American Psychiatric Association and the American Psychological Association adopted Position Statements absolutely prohibiting their members from participating in torture under any and all circumstances, and, to a limited degree, forbidding involvement in interrogations. Some interrogations utilize very aggressive techniques determined to be torture by many nations and organizations throughout the world. This paper explains why psychiatrists and psychologists involved in coercive interrogations violate the Geneva Conventions and the laws of the United States. Whether done with ignorance of professional ethical obligations or not, these psychiatrists and psychologists have crossed an ethical barrier that may best be averted from re-occurring by teaching medical students and residents in all medical specialties about the ethics principles stemming from the 1946–1947 Nuremberg trials and the Geneva Conventions, together with the Ethics Codes of the World Medical Association and the American Medical Association; and, with regard to psychiatric residents and psychological trainees, by the teaching about *The Principles of Medical Ethics With Annotations Especially Applicable to Psychiatry *and the *Ethical Principles of Psychologists and Code of Conduct*, respectively. In this way, all physicians and psychologists will clearly understand that they have an absolute moral obligation to "First, do no harm" to the human beings they professionally encounter.

## Background

In June 2005 the Board of Directors of the American Psychiatric Association (AΨA)^a^, concerned about allegations that psychiatrists participate in detainee interrogations at the United States naval station at Guantánamo Bay, Cuba, as well as in Iraq and Afghanistan, directed a number of AΨA components to clarify ethical and professional boundaries and recommend guidelines for psychiatrists' conduct in such settings [1]. After much discussion and debate regarding relevant issues, the AΨA, on May 21, 2006, approved a Position Statement, "Psychiatric Participation in Interrogation of Detainees," [2] declaring that no psychiatrist should participate directly in interrogation of prisoners^b^. News of the AΨA's action spread quickly; the Position Paper was widely acclaimed^c^.

At the same time, news media reported strong criticism of the American Psychological Association (APA) for permitting participation of psychologists in interrogation of detainees. Many psychologists resigned from the APA in 2007 [3] when the Board of Directors, disregarding the protest^d ^of a large number of members, accepted the 2005 recommendation of the Presidential Task Force on Psychological Ethics and National Security (PENS) that had been appointed to develop a position concerning the matter of psychologist participation in interrogations [4].

## Discussion

### PENS

PENS describes a clearly delineated role for psychologists "at distant and sequestered detention centers" created in the interest of national security and protection of the American people. According to Dr. Beth Shinn, the nine members of PENS included active-duty military officers and psychologists who worked for Defense Department agencies [5]. Some of these members were "in the chain of command at the places and during the times that abuses have been documented" [6].

We believe that even if the APA had adopted the same Position Statement as the AΨA, the existing interrogation programs involving psychologists would likely have continued unchanged. National obligations do not absolve clinicians of their primary commitment that we use our skills and training to heal. This societal trust or compact should never waver so it is unfortunate that the APA voted down a motion to ban participation altogether in interrogations at detention centers like Guantánamo Bay [7] and to state that the role of psychologists in settings in which detainees are deprived of their human rights should be limited to providing psychological treatment [5] – unfortunate because it served to strengthen the erroneous belief of many that the APA supports participation of psychologists in torture and fed into the claim by the AΨA that psychiatrists adhere, in the area of prisoner interrogations, to higher moral standards than psychologists. The fact that members of the APA Council of Representatives (COR) adopted the resolution titled "2007 Reaffirmation of the American Psychological Association Position Against Torture and Other Cruel, Inhuman, or Degrading Treatment or Punishment and Its Application to Individuals Defined in the United States Code as 'Enemy Combatants'" [8] does not mean that members of the APA are prohibited from participating in interrogation of prisoners. This is so because the Government's position is that "enhanced interrogation techniques," or, as President Bush put it, "alternative set of procedures" [9], do not constitute torture [10]. With "aggressive" [11] interrogation of detainees at Guantánamo and at secret overseas sites [12] ongoing at present, we believe, for reasons mentioned below, that these interrogations continue to include participation by psychologists and psychiatrists, as well.

### APA Council of Representatives

If the PENS recommendations had first been presented to the APA Council of Representatives [COR] (comprising at least 168 members), it is likely that these recommendations would have been rejected for instead adopting a policy statement similar to the AΨA's, if not stronger, prohibiting psychologists from direct participation in interrogations. The COR, like the AΨA Assembly, is a lively debating society in which the views of many representatives are expressed with passion and conviction; it is made up of strong-minded people selected by their APA branches to represent their views [13]. The COR, incidentally, has vastly more power than the AΨA Assembly. It is the legislative body of the APA and has full power and authority over the affairs and funds of the Association, including the power to review, upon its own initiative, the actions of any board, committee, division, or affiliated organization. The COR is APA's major legislative and policy setting body. Many APA members were greatly upset [13], not only because the COR was bypassed^e^, but because the APA PENS position was felt to approve what to them was clearly unethical conduct.

### AΨA Assembly

The AΨA Assembly, in comparison with the COR, has, as mentioned, very little power. It serves as the representative legislative component of the AΨA. Consisting of some 250 members, of whom approximately 160 are voting members (119 representatives and an equal number of non-voting deputy representatives are elected from the District Branches), the Assembly represents the members of the Association and acts for them in the affairs of the Association. Additionally, it provides a means for representing the needs of District Branches and for reciprocal communication between the District Branches and other governing bodies of the Association [14]. It is comprised of representatives from the Association's district branches who are elected by the members of the district branches. They advocate for their primary and special interests within the AΨA Central Office. Only in recent years has the AΨA Board of Trustees yielded to the Assembly's demand that all policy decisions of the Board be submitted for approval by the Assembly. It almost always approves the Board's actions. On rare occasions, it submits a minor amendment, which the Board usually accepts. More important, however, Action Papers (resolutions) proposed by Assembly members or District Branches and approved by the Assembly, by majority vote, and then submitted to the Board of Trustees, are referred by the Board to the Joint Reference Committee (JRC). The JRC frequently makes changes in the resolutions and passes them to the Board for adoption, without giving the Assembly the opportunity to review the altered resolutions.

### The amendment of the position statement

The amendment^f ^[15] to the position statement on psychiatric participation in interrogation of detainees, approved by the AΨA Assembly at its Nov. 2005 meeting, completely revising the third paragraph of the Position Statement, was *not *a minor amendment that the Assembly could just anticipate receiving automatic acceptance from the Board of Trustees. The Assembly had changed the wording of the third paragraph from "Psychiatrists should not participate in the interrogation of persons held in custody by military or civilian investigating or law enforcement authorities, whether in the United States or elsewhere. Nor should they provide information or advice to military or civilian investigative or law enforcement authorities regarding the likely consequences of specific techniques of interrogation that is in any way particularized in its application to an individual detainee" to "Psychiatrists should not participate in or assist any *coercive *interrogation of persons held in custody by military or civilian authorities, whether in the United States or elsewhere. Nor should they provide information or advice to military or civilian investigative or law enforcement authorities regarding the likely medical consequences of specific coercive methods. For purposes of this statement, *coercive *methods of interrogation include degradation, threats, isolation, imposition of fear, humiliation, sensory deprivation or excessive stimulation, sleep deprivation, exploitation of phobias, or intentional infliction of physical pain such as use of prolonged stress positions" (Emphasis added).

If these recommendations of the Assembly were accepted, the original and correct intent of the Board against any involvement in interrogations would be dangerously undermined: it would mean that the AΨA approved of participation by psychiatrists in "non-coercive" interrogations because now only the "coercive" interrogations would be off-limits. President Bush has authorized that the CIA and other agencies dealing with detainees can turn to "enhanced" techniques (i.e., coercive methods) that in the same double-speak are labeled "non-coercive." Such methods include waterboarding^g^, stress positions, use of military working dogs to instill fear, prolonged bombardment with loud music and flashing lights, deprivation of food, infliction of severely cold and hot temperatures, extreme sensory deprivation, shaking, striking, prolonged sleep deprivation, and isolation^h ^[11,16,17]. In 2006, George Annas (Professor and Chair, Department of Health Law, Bioethics, and Human Rights, Boston University) concluded that when doctors respond to detainees' hunger strikes by using the "'restraint chair' for 'postfeed observation' during which the prisoner must urinate and defecate on himself or herself, seems designed more for humiliation and subjugation than for medical treatment" [18].

It is quite concerning that the Assembly-amended version of the Statement did not mention most of these additional President-sanctioned coercive methods. According to the Pentagon's Office of the Inspector General (OIG), these techniques were transformed, with the assistance of military psychologists, into "standard operating procedure" (SOP) for interrogations at the Guantánamo Bay detention facility. This Guantánamo SOP, the OIG asserts, also was brought to Afghanistan and Iraq and, according to media reports, provided a basis for techniques used by CIA personnel, also with assistance from psychologists [19]. These techniques were designed to inflict physical and psychological harm for the purpose of breaking down interrogation subjects. The OIG report describes the nature and extent of that harm and the legal consequences to interrogators of employing techniques that cause it. Despite the euphemism "enhanced interrogation techniques," the International Committee of the Red Cross [20], the United Nations [21], Human Rights First and Physicians for Human Rights [22], and many others, classify them to be torture and also consider the techniques to be ineffective. Thus, the enhanced interrogation methods approved by the U.S. Government are in violation not only of Common Article 3 of the Geneva Conventions [23] but of at least three United States laws [24-26]. Interestingly, the British "no longer rely on assurances by the United States that it does not torture terrorism suspects" [27].

Despite the formal positions of both the APA [28] and AΨA [29] against torture, We believe that some psychologists and psychiatrists continue their participation in "enhanced interrogation techniques" authorized by the President of the United States [30]. Since this Presidential Order does not specify what techniques the CIA can and cannot lawfully engage in, it is reasonable to assume that the "enhanced" techniques authorized by the Secretary of Defense in March 2002 are still in use^i^. Unbelievably, a document signed, on August 1, 2002, by the head of the U.S. Justice Department's Office of Legal Counsel asserts "no method was torture unless it produced pain equivalent to organ failure or death"^j ^[31,32].

### The ticking time-bomb scenario

With regard to the ticking-time-bomb situation, Dr. Griffin Trotter [33] strongly supports the use of torture on a detainee believed to possess information concerning, say, a nuclear device that is about to be activated with the likely result that author and everyone else in half the world would be eliminated after suffering horribly painful irradiation burns ("In certain cases," he states, "the refusal to use torture would be morally reprehensible"). To this, we responded [34]: "This simplistic ticking time-bomb example, in one form or another, has been postulated over the centuries. It is mischievous, if not dangerous, not to mention beyond acceptable legal, moral, and ethical bounds; and exemplifies, with the current U.S. Government, even at the very highest levels, present-day justification to torture individuals suspected of possessing information that might benefit a particular cause, however seemingly noble or malignant that cause might be. Once one accepts this suggestion to violate laws against torture, the shameful journey on the slippery slope has begun. The absurdity of the ticking-bomb example is its presumption that the prisoner (terrorist) is a naive person who cannot figure out how to lie or who had not undergone the training necessary to deal with such a situation." In fact, in the almost 50 years since the ticking time-bomb scenario was first mentioned [35] there has not been a single occasion when such an incident was experienced.

Trotter is not alone in this readiness to accept torture in extreme situations. Alan Stone, M.D., a Harvard professor of law and psychiatry and past president of the American Psychiatric Association, is reported to have said: "I am unprepared to say that in a situation of great consequence, a psychiatrist who might have some ability to help our military should claim some ethical obligation that transcends all other obligations. There could come a time when I thought a person knows something and I could help find out what that is, I would certainly think it strange for me to rest on an ethical principle when there is so much greater harm [at stake]" [36]. Many others have called for torture in such situations; including Alan Dershowitz (a famous Harvard professor of law) [37], a distinguished judge of the United States Court of Appeals for the Seventh Circuit [38]; and a well-known forensic psychiatrist [39].

### The provenance of the AΨA Position Statement

#### a) The delay

Contrary to what is generally believed, adoption of the AΨA Position Statement was delayed by about six months, and did not receive unanimous approval by either the AΨA Assembly or Board of Trustees. Before describing how the Board of Trustees came to the decision to retain the Position Statement as originally drafted, it is important to further examine why the Assembly voted to so drastically amend the Statement. The then Speaker of the Assembly offered the following explanation (Joseph Rubin Email to [Assembly] Area Council members 02/16/2006): "During the November 2005 Assembly meeting, the Assembly amended the position statement, using terminology opposing psychiatric participation in *coerciv*e interrogation. It was pointed out at that time that military psychiatrists from the Society of Uniformed Services Psychiatrists are especially supportive of this language because they felt that the Board's language was too restrictive and could hamper their efforts to help the government and detainees. Some Assembly members also believe that there is no evidence indicating that any physician has been involved in the degrading aspects of interrogation."

#### b) The "forensicist"

Yet there is a much older culprit that led to the majority of Assembly members to vote as they did and it stems from the fact that many of them are engaged in forensic psychiatric practice in addition to their regular psychiatric activity (at least 13 representatives are in the full-time practice of forensic psychiatry). They, along with very many forensic psychiatrists in the United States, have been convinced that when they do forensic work, they are not required or expected to abide by all the canons in the code of medical ethics. This position was taken as a result of the very strong stand [40] expounded by one of this country's foremost experts in psychiatry and the law, Paul S. Appelbaum, M.D., formerly the A. F. Zeleznik Distinguished Professor and Chairman, Department of Psychiatry, University of Massachusetts Medical School; currently Elizabeth K. Dollar Professor of Psychiatry, Medicine and Law, and Director of the Division of Psychiatry, Law and Ethics, College of Physicians and Surgeons of Columbia University; past president of the American Psychiatric Association; and past president of the American Academy of Psychiatry and the Law. Appelbaum's position was forcefully endorsed by Thomas G. Gutheil, M.D., another leading figure in forensic psychiatry [41]. "The forensic psychiatrist," Appelbaum writes, "in truth does not act as a physician... If the essence of the physician's role is to promote healing and/or to relieve suffering, it is apparent that the forensic psychiatrist operates outside the scope of the role... Were we to call such a person a 'forensicist,' or some similar appellation, it might more easily be apparent that a different–nonmedical–role with its own ethical values is involved... [P]sychiatrists operate outside the medical framework when they enter the forensic realm, and the ethical principles by which their behavior is justified are simply not the same"^k ^[40]. "Their functioning in the forensic setting ... is guided by a different set of principles" [42]. Thus, by sanctioning the participation of psychiatrists in non-coercive interrogations, the slippery slope of clearly unethical involvement in this regard is created; for example, a policy recommended by a past president of the American Academy of Psychiatry and the Law under which participation by forensic psychiatrists in police interrogation of individuals who might be dangerous would not violate professional ethics, because such interrogations "are derivative of agency and efficacy, not ethics" [43]. This forensic psychiatrist also states: "Police interrogation techniques not only allow, but also encourage deception" [43]. Dr. Jeffrey Janofsky captures the essence of the problem succinctly: "Police interrogators routinely use deceptive techniques to obtain confessions from criminal suspects. When a psychiatrist directly uses, works with others who use, or trains others to use deceptive or coercive techniques to obtain information in police, military, or intelligence interrogations, the psychiatrist breaches basic principles of ethics" [44].

Dr. Appelbaum sees the forensic psychiatrist as an "advocate of justice" [40]. On the other hand, not only forensic psychiatrists, but all psychiatrists, must remain constantly alert to the danger of being drawn into unethical conduct in the service of an elusive and not infrequently unjust "justice" [45]. It has long been recognized that in countries where misuse of psychiatry has been, and in some countries still is, rampant, such as the former Soviet Union [46,47], China [48-53], Romania [54], South Africa [55] and others, psychiatrists justified their unethical conduct on the grounds that they were furthering the interests of their countries' justice.

It is regrettable, but understandable, that the influential military and forensic psychiatrists in the AΨA Assembly would agree with and endorse the view of a renowned forensic psychiatrist that "The theoretical framework offered by Appelbaum has become a mainstay of our understanding of forensic ethics" [56]. It should be recognized, however, that Appelbaum bases his entire argument that a special code of ethics is necessary for forensic psychiatrists on his mistaken belief that AΨA's *Principles of Medical Ethics With Annotations Especially Applicable to Psychiatry *is restricted to the physician-patient relationship in which "the physician's role is to promote healing and/or to relieve suffering" [57]. Rather than needing a special set of ethics for the forensic psychiatrist, the principles and annotations of the ethics code (2001 Edition) is for all psychiatrists. As examples; Principle #1 ("A physician shall be dedicated to providing competent medical service with compassion and respect for human dignity"); Principle #3 ("A physician shall respect the law and also recognize a responsibility to seek changes in those requirements which are contrary to the best interests of the patient"); Principle #7 ("A physician shall recognize a responsibility to participate in activities contributing to an improved community"); Annotations #6 of Section 4 ("Psychiatrists are often asked to examine individuals for security purposes, to determine suitability for various jobs, and to determine legal competence. The psychiatrist must fully describe the nature and purposes and lack of confidentiality of the examination to the examinee at the beginning of the examination"); Annotation #8 of Section 4 ("Psychiatrists at times may find it necessary in order to protect the patient or the community from imminent danger, to reveal confidential information disclosed by the patient"). Appropriate *guidance *to assist forensic psychiatrists in adhering to the code of medical ethics is provided in the *American Academy of Psychiatry and the Law Ethics Guidelines for the Practice of Forensic Psychiatry *(adopted May 2005). Thus, we unreservedly disagree with Appelbaum's position that "forensic psychiatrists cannot simply rely on general medical ethics" [57]. General medical ethics have considered the ethics of physician participation in interrogations and clearly forbid it. Now is the time for all clinicians to live up to the existing codes rather than attempt to weaken them.

#### c) The path to adoption of the AΨA Position Statement

We now describe in greater detail the events that surrounded the adoption of the final version of the AΨA Position Paper on Psychiatric Participation in Interrogation of Detainees.

The AΨA Board of Trustees approved their initial proposed Position text on October 10, 2005. Soon after, we warned Steven S. Sharfstein, M.D., the then president of the AΨA, that "there are some 'forensicists' who will try to scuttle it [the Position Statement] by amendment or tabling" it when it is presented to the Assembly for their approval at their meeting on November 11–14, 2005 (A.L.H. Email to Steven Sharfstein 11/02/2005). Dr. Sharfstein thanked us for alerting him (Steven Sharfstein Email to Abraham Halpern 11/02/2005). As mentioned above, the Assembly added the word "coercive" [58], thereby sanctioning the participation of psychiatrists in "non-coercive" interrogations. Should this language be retained in the final Position Statement, given the Government's definition of non-coercive interrogation techniques, psychiatrist participation in what in actuality constitutes coercive techniques would instead be viewed as meeting with the Board's approval. Moreover, it would open the door for psychiatrists to engage in deceptive practices, already declared, as noted above, to be a matter of "agency and efficacy, not ethics" [43]. Since the Board would be meeting on December 11–12, 2005, less than four weeks after the Assembly adopted its amended Position Statement, we decided to contact as many members of the Board as possible to alert them to the importance of retaining the original Position Statement. Dr. Sharfstein was notified (A.L.H. Email to Steven Sharfstein 11/26/2005) that accepting the changes recommended by the Assembly would result in sanctioning the misuse of psychiatry in interrogations of detainees, such as the use of deception to elicit information with the aid of psychiatrists. Dr. Sharfstein thanked us (Steven Sharfstein Email to A.L.H. 11/28/2005). Alfred M. Freedman, M.D., emeritus professor and chairman, Department of Psychiatry, New York Medical College who is also a member of the AΨA Board of Trustees (and with whom we had collaborated on a number of related topics over the previous 10 years [59,60]), was kept informed of our correspondence. Dr. Sharfstein was provided additional reasons to reject the Assembly's amendment (many of which are included in the "Forensicist" section above) (A.L.H. Email to Steven Sharfstein 11/29/2005). We also notified Herbert Peyser, M.D., a long-serving member of the Assembly, and Michael Blumenfield, M.D., Speaker-Elect of the Assembly, of the potential dangers if the Assembly's final Position Statement became AΨA policy (A.L.H. Email to Herbert Peyser and Michael Blumenfield 11/30/2005). Dr. Freedman wrote a lengthy letter to Dr. Sharfstein strongly urging him to do everything he can to obtain the Board's approval to reject the Assembly's amendments and emphasizing the likelihood that psychiatrists attending "non-coercive" interrogations would facilitate interrogators' use of techniques that are in violation of both the Geneva Accords and U.S. laws (Alfred Freedman Email to Steven Sharfstein 11/0/2005). Dr. Sharfstein fully agreed with Dr. Freedman and asked permission for Dr. Freedman's "note be distributed to the Board" for discussion at the Board meeting on December 10–11 (Steven Sharfstein Email to Alfred Freedman 12/01/2008). Other members of the Board were sent additional messages about the implications of retaining the Assembly's ammendments (A.L.H. Email to Lawrence Hartmann 12/03/05; A.L.H. Email to Pedro Ruiz 12/03/05; A.L.H. Email to Harold Eist 12/05/05; A.L.H. Email to Carolyn Robinowitz, Nada Stotland, Michelle Riba, Marcia Goin and Ann Sullivan 12/07/05; A.L.H. Email to Alan Stone 12/10/2005).

A few days prior to the AΨA Board meeting, the Board received a letter from Leonard Rubenstein, J.D., the Executive Director of Physicians for Human Rights (PHR), in essence recommending that the AΨA Position Statement "be limited to interrogations outside the U.S. justice system (Leonard Rubenstein Letter to Steven Sharfstein and members of the APA Board of Trustees 12/07/2005). Such a change would still result in the Position Statement exempting participation by psychiatrists in interrogations by police officials and other authorities within the United States, and, as such, could pose a serious risk of misuse and abuse of psychiatry. We wrote to Dr. Sharfstein and several members of the Board of Trustees, including Dr. Paul Appelbaum, on December 8, strongly urging them not to accept the suggestion of PHR (A.L.H. Email to Steven Sharfstein and several Board members 12/08/2005). We received a positive response from Dr. Appelbaum indicating that he would do what he could to see that our recommendation was accepted by the Board (Paul Appelbaum Email to A.L.H. 12/08/2005). On December 9, Mr. Rubenstein, wrote to Dr. Freedman indicating that PHR had intended only to suggest a compromise in the event that the differences in the positions of the Board and the Assembly could not be resolved, and that PHR was withdrawing its suggestion and was entirely supportive of the Board's original Statement unchanged (Leonard Rubenstein Email to Alfred Freedman 12/09/2005).

At the Board of Trustees meeting, although some members opposed the original Position Statement, the Board voted unanimously "to reaffirm the position statement approved at the October 2005 Board meeting, with the understanding that the Board will initiate a dialogue with Assembly leaders and with as many Assembly members as possible before the May 2006 Assembly meeting to approve a joint statement." The AΨA President and Speaker were to appoint Board and Assembly members representing both sides of this issue to an ad hoc work group with a charge to propose recommendations in time for the May 2006 Assembly and Board meetings (Joseph Rubin Email to [Assembly] Area Council members 02/16/2006). The purpose of this committee was to see if some revision of the Statement could be developed that would accommodate both the Board's and Assembly's versions of the Position Statement. The position of the military and forensic psychiatrists in the Assembly was clearly presented; nevertheless, although the committee members were not unanimous, the committee voted to recommend to the Assembly that the Board's original Position Statement on Psychiatric Participation in Interrogation of Detainees become official AΨA policy. The position (fully consistent with the "forensicist" views discussed above) of the forensic psychiatry and military Assembly representatives, strongly supportive of the amendment to limit the prohibition of psychiatrist participation to "coercive" interrogations, was well summarized by Joseph Rubin, M.D., as follows (Joseph Rubin (Speaker of Assembly) Email to James Nininger (Speaker-Elect) and Thomas Grieger (Representative of the Society of Uniformed Services Psychiatrists) 02/17/2006):

The Assembly representatives from the work group agreed that the revised document narrows the differences between the previously stated positions and broadens the potential role of psychiatrist participation. However, the Assembly representatives believe that there may be benefit to psychiatrists advising on non-coercive interview techniques related to particular detainees. For forensic psychiatrists, the primary value of their work is to advance the interests of justice. Trying to introduce the principles of medical ethics for treatment into a theory of ethics for forensic psychiatry heightens the problem of dual agency. When serving the interests of justice, forensic psychiatrists must adhere to the general moral rules of disclosure and respect for patients. If the role of the psychiatrist is made clear, and there are prohibitions against cruel, inhuman, or humiliating conditions, psychiatrists consulting to interviewers of detainees can adhere to general ethical principles. Enhanced gathering of information may assist in the pursuit of justice, identification of other dangerous individuals, or the curtailment of future acts against society.

Forensic psychiatrists routinely provide direct examination of individuals charged with crimes for purposes other than providing treatment (examples include assessing competency to stand trial and possible lack of criminal responsibility due to psychiatric illness). Psychiatrists also assist defense counsel in working with distrusting clients, prosecuting attorneys examining witnesses during trial and law enforcement or correctional authorities gathering information from suspects, crime witnesses, or prisoners with behavior problems. Suggestions for interviewers in these settings (or in the case of detained individuals) are similar in many ways to the supervision and training we provide to psychiatry residents and medical students as they develop their interviewing skills. These suggestions might include the pacing and phrasing of questions, observation of non-verbal cues provided by the person being questioned, selection of an alternative interviewer, and changing the length or frequency of interview sessions.

Very apprehensive that, notwithstanding the ad hoc committee's recommendation, the AΨA Assembly might persist in their efforts to amend the Position Statement (and thus create a dispute between the Assembly and Board of Trustees that would be very embarrassing for the AΨA), we urged Dr. Appelbaum to make a presentation to the Assembly when it took up the matter of the Position Statement again at its meeting on May 21, 2006 (A.L.H. Email to Paul Appelbaum 05/17/2006). Dr. Appelbaum, as noted above, had already agreed with our strong objections to the amended Position Statement, notwithstanding the position of his fellow "forensicists" in the Assembly (Paul Appelbaum Email to A.L.H. 12/08/2005). Accordingly, as a member of the Board of Trustees and designated to represent the Board, he addressed the Assembly and explained the reasons for the Board's Position Statement, emphasizing the facts that "interrogations are inherently deceptive/coercive, and hence inappropriate for physicians to be involved" and "involvement undercuts the physician's role as treater, because detainees are unlikely to trust physicians when they know that they or their colleagues participate in interrogations"^l^. This, apparently inadvertently overlooked by Dr. Appelbaum, negates his assertion, noted in "The Forensicist" section above, that "The forensic psychiatrist in truth does not act as a physician."

In the end, the Assembly, by a majority vote, approved the Board of Trustees' original Position Statement on Psychiatric Participation in Interrogation of Detainees, which became official AΨA policy as of May 21, 2006. (Some members of the Assembly and at least one military psychiatrist, although speaking in favor of the original Statement, urged the Assembly to somehow change the Statement to take into consideration the unique position the Statement imposes on those physicians assigned to assist interrogation personnel).

## Conclusion

We believe that the *AΨA Position Statement on Psychiatric Participation in Interrogation of Detainees *does not justify the AΨA's presenting itself to the world as an organization adhering, in the matter of interrogation of detainees using techniques condemned by all civilized nations, more faithfully to principles of ethics than the APA. A relatively small number of psychologists and psychiatrists, no doubt, like forensicists, are convinced that they operate "outside the bounds of the doctor-patient relationship and [are] thus not required to abide by accepted ethical guidelines" [61] and continue to participate in interrogation of prisoners in military and CIA detention centers. They do so in the belief that they are "doing their duty" to uncover terrorist threats against the United States, whether the interrogation procedures are "enhanced" or not, and that their presence at interrogations protects detainees from "drifting" of interrogators beyond authorized enhanced techniques [62]. There is no doubt that participation in interrogations of arrestees charged with crime in this country is a far more frequent occurrence. Here, their justification is, mistakenly, that, as "forensicist" psychiatrists and psychologists, they are expected to adhere to an ethics code that is different from the long-established codes of the AΨA [63] and the APA [64], respectively. In our view, these psychologists and psychiatrists, even when they (having been led to believe that the prisoners have vital information about planned terrorist attacks against the United States) are heavily involved in "enhanced" interrogation procedures that are clearly in violation of the Geneva Conventions and the laws of the United States, would not be subject to disciplinary action by an ethics committee. Given that they would have acted in compliance with what they believe was legally authorized by the President of the United States or the Secretary of Defense, no state licensing authority is likely to investigate such government-sanctioned participation in a time of war. Regardless of the differently worded position statements of the APA and AΨA, there is no difference in the end result of some psychologists and psychiatrists continuing to participate in interrogations of detainees where a threat to national security or danger of terrorist attack is believed.

It is only by teaching medical students, residents in all medical specialties, and psychology trainees and interns about the ethics principles stemming from the Nuremberg trials [65] and the Geneva Conventions [23], together with the Ethics Codes of the World Medical Association and the American Medical Association; and, with regard to psychiatric residents and psychological trainees, by the teaching about *The Principles of Medical Ethics With Annotations Especially Applicable to Psychiatry *and the *Ethical Principles of Psychologists and Code of Conduct*, respectively, that all physicians and psychologists will understand that they have an absolute moral obligation to "First, do no harm" [66]. It is a separate matter as to whether ongoing participation in interrogations by some psychologists and psychiatrists will result in professional sanction through official regulatory agencies or professional societies. Whether these psychologists and psychiatrists believe they are contributing to "national security" by their actions or whether or not they justify their actions by claims of Presidential approval, the damage to our professions remain the same: doctors are abusing their training in service to country at the expense of the interrogated individual. With further education and wider circulation of the Position Statements of the AΨA and APA, we may prevent the next generation of caregivers from continuing this disgraceful and unethical misapplication of medical and mental health knowledge.

### Endnotes

a) We do not wish to cause any confusion with abbreviations, so we use the Greek character Psi (which is widely used to represent "Psychiatry") in our abbreviation for the American Psychiatric Association: "AΨA", and "APA" for the American Psychological Association.

b) The relevant part of the Statement is the third paragraph which reads as follows:

"3. No psychiatrist should participate directly in the interrogation of persons held in custody by military or civilian investigative or law enforcement authorities, whether in the United States or elsewhere. Direct participation includes being present in the interrogation room, asking or suggesting questions, or advising authorities on the use of specific techniques of interrogation with particular detainees. However, psychiatrists may provide training to military or civilian investigative or law enforcement personnel on recognizing and responding to persons with mental illnesses, on the possible medical and psychological effects of particular techniques and conditions of interrogation, and on other areas within their professional expertise."

c) Unfortunately, the strength of the Position Statement was significantly weakened when, immediately following the approval of the Statement by the Assembly on May 21, 2006, Steven S. Sharfstein, M.D., the then president of the AΨA, made comments in response to a reporter's questions, reported as follows: "Dr. Sharfstein acknowledged that psychiatrists in the military might have a conflict between obeying the AΨA's policy and following direct orders, noting the position statement is not 'an ethical rule.' 'Individual psychiatrists wouldn't get in trouble with the AΨA' for failing to follow the guidelines, he said. 'If they're given an Army order, that would be another question,' Dr. Sharfstein said. Currently, he said, there are only a 'few psychiatrists who are participating in the interrogations. That was not true in the past"' [67]. The impact of this comment is not lessened by a subsequent statement he is reported to have made following an announcement by the Defense Department that military psychiatrists were not "ordinarily" to be used as consultants to interrogators, "but may be so assigned" when psychologists are unavailable: "Those circumstances do not exempt that psychiatrist from AΨA's statement. There are no exceptions" [28]. Nevertheless, it remains abundantly clear that the need for involvement of psychiatrists in interrogations, as perceived by the Defense Department, trumps the prohibition by the AΨA Statement–and there are exceptions.

d) These protests have increased markedly to the point where a major challenge to the APA policy will take place at the August 2008 annual meeting in Boston [68].

e) The APA Board of Directors arbitrarily approved the PENS recommendations without obtaining COR consent on the grounds that an "emergency" existed [6]. The Board of Trustees of the AΨA, on the other hand, referred its Position Statement to the Assembly and did not automatically declare it to be AΨA policy.

f) The relevant part of the amended Statement is the third paragraph, which reads as follows: "3. No psychiatrist should participate in or assist any coercive interrogation of persons held in custody by military or civilian authorities, whether in the United States or elsewhere. Nor should they provide information or advice to military or civilian investigative or law enforcement authorities regarding the likely medical consequences of specific coercive methods. For purposes of this statement coercive methods of interrogation include degradation, threats, isolation, imposition of fear, humiliation, sensory deprivation or excessive stimulation, sleep deprivation, exploitation of phobias, or intentional infliction of physical pain such as use of prolonged stress positions."

g) Although, as reported in The New York Times, waterboarding involves strapping a suspect in a board with feet elevated, covering his face with a cloth and pouring water on it to produce a feeling of suffocation (mock drowning) [12], waterboarding is almost always referred to as "waterboarding" without any elaboration in the psychiatric and psychological literature. Christopher Hitchens gives a more vivid description [69] after volunteering to go through the experience with a team of SERE veterans in North Carolina. He states, in part: "You may have read by now the official lie about this treatment, which is that it 'simulates' the feeling of drowning. This is not the case. You feel that you are drowning because you *are *drowning–or, rather, being drowned, albeit slowly and under controlled conditions and at the mercy (or otherwise) of those who are applying the pressure. The 'board' is the instrument, *not *the method. You are not being boarded. You are being watered. This was very rapidly brought home to me when, on top of the hood, which still admitted a few flashes of random and worrying strobe light to my vision, three layers of enveloping towels were added. In this pregnant darkness, head downward, I waited for a while until I abruptly felt a slow cascade of water going up my nose. Determined to resist if only for the honor of my navy ancestors who had so often been in peril on the sea, I held my breath for a while and then had to exhale and–as you might suspect–inhale in turn. The inhalation brought the damp cloths right against my nostrils, as if a huge, wet paw had been suddenly and annihilatingly clamped over my face. Unable to determine whether I was breathing in or not, and flooded more with sheer panic than mere water, I triggered the pre-arranged signal and felt the unbelievable relief of being pulled upright and having the soaking and stifling layers pulled off me." " [I]f," Hitchens adds, "waterboarding does not constitute torture, then there is no such thing as torture."

h) These techniques were based in large part on techniques of torture and cruelty used by the U.S. military in its Survival, Evasion, Resistance, and Escape (SERE) program. The SERE program, itself "based on Chinese techniques used in the 1950s that produced false confessions from American prisoners" [68], was intended to train pilots, special forces, and other potential high-value captives against torture, should they be captured by a power that does not respect the Geneva Conventions [70,19]. The SERE program "rests on the belief that inflicting a controlled level of pain and humiliation on those who might face it in combat would help them survive the real thing if they were captured. For the C.I.A. after 2001, SERE became not a tool for resisting torture, but a template for inflicting it–a template soon adopted by interrogators in the far-flung 'black sites' where detainees were imprisoned" [71,72]. "Behavioral scientists" took part in the training of interrogators in the use of SERE "aggressive" techniques of interrogation [11].

i) Later in 2002, the "Behavioral Science Consultation Team" (BSCT) was created at Guantánamo in order to develop new strategies for interrogation and assess intelligence production [73,74]. The staff included a psychiatrist and a psychologist [75]. The "strategies" [76], approved by Justice Department and National Security Council lawyers (and over the secret objections of every branch of the military services and the Federal Bureau of Investigation) had, for decades prior to 2002, been judged by the United States to be illegal torture [11].

j) This opinion defining torture was ostensibly withdrawn two years later; however, a Justice Department spokesman has stated, according to an Associated Press report [77], that "the conclusions of the opinion approving specific interrogation methods are still in force." That these methods continue to be used is, in fact, confirmed by the June 17, 2008, report of the Senate Armed Services Committee Hearing [11].

k) This brings to mind the comment by George Annas [31]: "Bloche and Marks have reported, on the basis of their interviews with some of the physicians involved in interrogations at Guantánamo Bay and in Iraq, that the physicians believed 'that physicians serving in these roles do not act as physicians and are therefore not bound by patient-oriented ethics"' [78].

l) Dr. Appelbaum's persuasive comments are noted in a memorandum to the Speaker just prior to the stormy session in the Assembly about the Position Statement: "Why should psychiatrists not advise interrogators on strategies to take with particular detainees? Interrogations, even when conducted legally, are inherently coercive and deceptive; coercive because a detainee who wanted to talk would not have to be interrogated; deceptive because interrogators are trained to mislead suspects and are supported by the law in doing so. The purpose of an interrogation is to pressure or trick the detainee into revealing information that the detainee does not want to disclose. These may be legitimate functions for law enforcement. But psychiatric ethics–even as applied in forensic evaluations, which may be the closest analogue–require us to obtain consent before questioning a person, and preclude our lying to them to obtain information. When forensic psychiatrists conduct an interview, the purpose is to assess the person's mental state. Here, however, the purpose of involvement is to extract information, and the psychiatrist's job is to guide interrogators in doing so. Having psychiatrists so closely allied with the policing function of the state threatens to compromise our healing mission. That most people consider this to be an inappropriate role for any physician seems apparent from the public reaction to recent allegations of physician involvement in interrogations. Our profession would be best served if we too recognized the inappropriateness of our playing this role." It is clear to the authors of this article that Dr. Appelbaum by his statement, particularly his reference to "the inappropriate role for any physician," has failed to see that, by extrapolation, his words can apply to the "forensicist." It is hoped that forensic psychiatrists will now understand that a special ethics code for forensic psychiatrists is unwarranted, and that they, along with all other psychiatrists, are honor bound to abide by *The Principles of Medical Ethics With Annotations Especially Applicable to Psychiatry*.

## Competing interests

The authors declare that they have no competing interests.

## Authors' contributions

ALH conceived this paper's topic, and all authors participated in the coordination of reviewed materials and helped draft the manuscript. All authors read and approved the final manuscript.

## Author information

Abraham L. Halpern M.D. is Emeritus Professor of Psychiatry at New York Medical College. He is a past president of both the American Academy of Psychiatry and the Law, and the International Academy of Law and Mental Health.

John H. Halpern M.D. is Assistant Professor of Psychiatry, Harvard Medical School; Director of the Laboratory for Integrative Psychiatry, Addictions Division, and Associate Director of Substance Abuse Research, Biological Psychiatry Laboratory, McLean Hospital.

Sean B. Doherty BSc. (Hons) is a fifth year medical student at Cardiff University (as part of the Swansea University GEP medicine course). He received the 2008 Royal College of Psychiatry Medical Elective Bursary, which helped fund his current two-month placement as a research assistant at the Laboratory of Integrative Psychiatry, Addictions Division, McLean Hospital, Belmont, MA.
